# The association between paternal depressogenic cognitive styles during pregnancy and offspring depressogenic cognitive styles: an 18‐year prospective cohort study

**DOI:** 10.1111/jcpp.12847

**Published:** 2017-11-24

**Authors:** Gemma Lewis, Siying Wen, Rebecca M. Pearson, Glyn Lewis

**Affiliations:** ^1^ Division of Psychiatry Faculty of Brain Sciences University College London London UK; ^2^ School of Social and Community Medicine University of Bristol Bristol UK

**Keywords:** Cognitive style, depression, paternal, adolescent, epidemiology, ALSPAC

## Abstract

**Background:**

Preventing the development of depressogenic or negative cognitive styles could also prevent the development of depression, a leading public health problem worldwide. Maternal negative cognitive styles are a modifiable risk factor for the development of negative cognitive styles in offspring. However, evidence on the role of paternal negative cognitive styles is inconclusive and there have only been a few small studies, which may also have lacked statistical power.

**Methods:**

We used data from the Avon Longitudinal Study of Parents and Children (ALSPAC) to investigate the association between paternal negative cognitive styles, measured when mothers were 18 weeks pregnant, and offspring negative cognitive styles 18 years later (*N* = 6,123). Associations were calculated using linear regression models, before and after adjustment for confounders including maternal negative cognitive styles. We compared associations before and after controlling for depression in parents and offspring, and used multiple imputation to reduce biases that may have arisen due to missing data.

**Results:**

A two‐standard deviation increase in paternal negative cognitive style was associated with a 3‐point increase in offspring negative cognitive style (95% CI 1.36–4.37). This association remained after adjustment for confounders and was independent of depression in both parents and offspring. The effect size was equivalent to that of maternal negative cognitive style, and was also independent of maternal negative cognitive style.

**Conclusions:**

Our results suggest that fathers should be included in individual‐ and family‐based interventions designed to prevent the development of depressogenic cognitive styles in adolescent offspring. This could possibly also prevent the development of depression.

## Introduction

The prevalence of depression is high in most countries, and there is some evidence that it is increasing (Ferrari et al., 2013; McManus, Bebbington, Jenkins, & Brugha, 2016). While there are effective ways of treating depression, there are no generally accepted methods of prevention. Most of the risk factors for depression involve stressful events or circumstances that are often outside of an individual's control. However, it might be possible to modify how people respond to the stressful events they experience, to promote resilience to stressors and so prevent depression.

According to Beck's cognitive theory of depression, negative thinking styles or ‘negative cognitive schemas’ play a key role in the development of depressive illness (Beck, 2008). Negative schemas are learned from prior experiences, and alter the interpretation of new information. For example, the belief or schema ‘I am a failure’ can lead to negative interpretations of new information, altering an individual's response to stressors and increasing their risk of depression. Beck's theory is similar to the helplessness or hopelessness theory of depression, which focuses on how people perceive the causes of events (Abramson, Seligman, & Teasdale, 1978; Alloy, Abramson, Metalsky, & Hartlage, 1988). People with a tendency to believe that they are the cause of an event (an internal attribution); that the event is difficult to change (a stable attribution); that it will lead to failures in other areas of their lives (a global attribution); and that it reflects their self‐worth, are at increased risk of depression (Seligman et al., 1984).

The theories by Beck and Abramson both propose a psychological or cognitive vulnerability to depression and the processes they describe, although not identical, are very similar (Haeffel et al., 2003). The negative schemas proposed by Beck are measured using the Dysfunctional Attitudes Scale (DAS), and causal attributions with the Cognitive Styles Questionnaire (CSQ). The DAS and CSQ have been found to correlate moderately to strongly (Pearson et al., 2014), and there is conceptual overlap as well. For example the DAS item ‘I always expect criticism’ can be assumed to reflect ‘stable’, ‘global’ and ‘self‐worth’ causal attributions measured by the CSQ. We will therefore refer to both concepts as a negative cognitive style (Pearson et al., 2013).

Negative cognitive styles are important because they can result in psychological vulnerability to depression. They are a major target for cognitive behavioural therapy (CBT), an effective psychological treatment for depression that modifies how people interpret and respond to emotional information (Cuijpers, Cristea, Karyotaki, Reijnders, & Huibers, 2016). Many population‐based cohort studies report that more negative cognitive styles increase risk of future depression in adults and adolescents (Alloy et al., 2006; Evans, Heron, Lewis, Araya, & Wolke, 2005; Hankin, 2008; Hankin & Abramson, 2002; Lakdawalla, Hankin, & Mermelstein, 2007; Pearson et al., 2014; Rawal, Collishaw, Thapar, & Rice, 2013). So, preventing the development of negative cognitive styles may also be one way of reducing the incidence and prevalence of depression.

Our previous study found that maternal negative cognitive styles, measured when mothers were 18 weeks pregnant, were associated with more negative cognitive styles in offspring 18 years later (Pearson et al., 2013). This association was independent of depression in both mothers and offspring. However, to our knowledge, only a few studies have investigated associations between paternal and offspring negative cognitive styles (Alloy et al., 2001; Kaslow, Rehm, Pollack, & Siegel, 1988; Oliver & Berger, 1992; Seligman et al., 1984; Stark, Schmidt, & Joiner, 1996; Turk & Bry, 1992). This is important to address, since fathers are increasingly involved in the care of their children in many countries (Kroll, Carson, Redshaw, & Quigley, 2016).

To our knowledge, the few studies of paternal and offspring negative cognitive styles have found limited evidence for an association (Alloy et al., 2001; Kaslow et al., 1988; Oliver & Berger, 1992; Seligman et al., 1984; Stark et al., 1996; Turk & Bry, 1992). However, these studies had various methodological limitations, which may have led to unreliable findings. First, sample sizes were often very small, ranging from 15 to 118 fathers. In our previous large study of maternal and offspring cognitive styles, we found that the association persisted over 18 years, but the effect size was small. Existing studies may have lacked statistical power to detect a similarly small effect size. Many of the studies also selected participants who exceeded cut‐off scores on measures of cognitive vulnerability or depression/anxiety or examined only university students, so might have introduced a selection bias. For example, one study focused only on students with academic problems (*N* = 21) (Turk & Bry, 1992). In addition, only one study of a student sample was longitudinal (Alloy et al., 2001).

In this study, we used a large population‐based birth cohort to investigate associations between paternal negative cognitive styles, measured during pregnancy, and offspring negative cognitive styles 18 years later.

## Methods

### Participants

The Avon Longitudinal Study of Parents and Children (ALSPAC) is an ongoing population‐based birth cohort examining a wide range of influences on health and development (Boyd et al., 2013). All pregnant women living in the former county of Avon in Bristol, South West England (UK), with an estimated delivery date between April 01 1991 and December 31 1992, were invited to participate. The core enrolled sample consisted of 14,541 women (an estimated 85%–90% of those eligible) and 13,154 fathers/partners. Of 14,062 live births, 13,798 were singletons or first‐born twins alive at 1 year of age. Since pregnancy, mothers, fathers and offspring have regularly provided data, either through postal questionnaires or by attending research clinics. Further information about ALSPAC is available on the study website (http://www.bristol.ac.uk/alspac), which includes a fully searchable data dictionary: (http://www.bris.ac.uk/alspac/researchers/data-access/data-dictionary). In this study, we used data from core ALSPAC singleton offspring whose fathers had provided data on their cognitive styles during the mother's pregnancy.

### Ethical approval

Ethical approval for the study was obtained from the ALSPAC Law and Ethics Committee and the Local NHS Research Ethics Committee. All participants provided written informed consent.

### Measures

#### Offspring negative cognitive styles

Adolescents completed the Cognitive Style Questionnaire Short Form (CSQ‐SF) at a research clinic when they were an average age of 17 years and 10 months. The CSQ‐SF was developed from the original CSQ which measures cognitive vulnerability according to the hopelessness theory of depression (i.e. causal attributions for events) (Haeffel et al., 2008). The CSQ‐SF was developed for adults but has been validated for use in older adolescents and has good psychometric properties (Meins et al., 2012)_._ The CSQ‐SF presents participants with eight hypothetical situations relating to failures in academic achievement, employment and interpersonal relationships. Participants are asked to place themselves in each situation and decide what they think would have caused that situation if it actually happened to them. After each situation, there are eight statements representing the possible causal attributions (internal, stable, global, self‐worth). Each attribution is represented by two statements, resulting in 64 items. Participants are asked to rate the extent to which they agree with each statement using a 5‐point Likert scale ranging from ‘strongly agree’ to ‘strongly disagree.’ For example, in response to the following situation ‘Imagine you are getting along badly with your parents’, adolescents rate the extent to which they agree with eight statements such as: ‘It is my fault if I am getting along badly with my parents’ (internal); ‘the reason I get on badly with my parents causes problems in all areas of my life’ (global); the reason for getting along badly will stop me from getting along well with my parents in the future’ (stable); ‘getting along badly with my parents means there is something wrong with me as a person’ (self‐worth). Possible total scores range from 64 to 320, with higher scores indicating a more negative cognitive style.

#### Paternal negative cognitive styles

A measure of paternal negative cognitive style was derived from ‘The Interpersonal Sensitivity Measure’ (IPSM), a self‐report questionnaire administered to parents when mothers were 18 weeks pregnant. The IPSM contains 36 items assessing a range of negative beliefs about the self (Evans et al., 2005). Six items that originated from the DAS were selected in a previous study, to measure negative cognitions as outlined in Beck's cognitive theory (Evans et al., 2005). In a prior study, these items were also selected independently by two authors, for inter‐rater reliability (Pearson et al., 2013). Items that included words directly related to mood such as ‘worry’ were excluded to ensure that a different construct to depression was being assessed. Participants rated the extent to which each of the six items described them using a 4‐point Likert scale ranging from ‘very like me’ to ‘very unlike me.’ Responses were summed to yield a total score ranging from 0 to 18, with higher scores indicating a more negative cognitive style. This measure was used in our previous study of maternal cognitive styles (Pearson et al., 2013).

#### Offspring depressive symptoms

Offspring depression was assessed in the same research clinic as the cognitive styles assessment (mean age 17 years, 10 months), using a computerized self‐administered version of the Clinical Interview Schedule‐Revised (CIS‐R) (Lewis, Pelosi, Araya, & Dunn, 2009). The CIS‐R derives diagnoses of depression based on ICD‐10 criteria and produces a continuous severity score by adding the depression, depressive ideas, concentration, sleep and fatigue items. In this study, we used the continuous score. Possible scores range from 0 to 21, with higher scores indicating more severe depressive symptoms. Depressive symptoms were also measured earlier in the study, using the short Mood and Feelings Questionnaire. The sMFQ is a 13‐item self‐report measure of the severity of DSM‐IV depressive symptoms occurring in the past 2 weeks (Angold et al., 1995). Possible scores range from 0 to 26, with higher scores indicating more severe depressive symptoms. We used the continuous sMFQ at age 16.

#### Potential confounders

We investigated as potential confounders variables that have previously been associated with negative cognitive styles. These included parent age, highest educational qualification, social class, smoking during the mother's pregnancy and depressive symptoms (Alloy et al., 2001; Kaslow et al., 1988; Oliver & Berger, 1992; Pearson et al., 2013; Seligman et al., 1984; Stark et al., 1996; Turk & Bry, 1992). Educational qualification was coded 1–5, ranging from Certificate of Secondary Education (1) which used to be compulsory in the United Kingdom, to university degree (5). This was dichotomized to create a binary variable (compulsory and noncompulsory education). Social class was measured using five categories from the 1991 classification of the UK Office of Population Censuses and Surveys, dichotomized into manual and nonmanual (Tilling, Macdonald‐Wallis, Lawlor, Hughes, & Howe, 2014). These variables were derived from the same questionnaire that assessed paternal cognitive styles at 18 weeks gestation. Paternal depressive symptoms were assessed using the Edinburgh Postnatal Depression Scale (EPDS), a 10‐item self‐report questionnaire designed to screen for symptoms of perinatal depression occurring in the past 7 days. The EPDS has been validated for use in men (Edmondson, Psychogiou, Vlachos, Netsi, & Ramchandani, 2010). Possible scores range from 0 to 30, with higher scores indicating more severe depressive symptoms. Scores greater than or equal to 12 have high sensitivity and specificity for detecting major depression (Carothers & Murray, 1990). We adjusted for continuous EPDS scores. We adjusted for the same characteristics in the mother and also for maternal negative cognitive styles, whether the parents lived together, and the sex of the child.

### Statistical analyses

#### The association between paternal and offspring negative cognitive styles, and the role of maternal negative cognitive styles

We used linear regression models to examine the association between paternal negative cognitive styles (continuous exposure) and offspring negative cognitive styles (continuous outcome). Offspring cognitive style scores were approximately normally distributed. For comparability with results already published on maternal cognitive style (Pearson et al., 2013), we examined a two‐standard deviation (6‐point) increase in the exposure. Our outcome remained unstandardized. We also present standardized beta coefficients in Table S1 First, we examined a univariable (unadjusted) model containing the exposure and outcome. Next, we added maternal negative cognitive styles as an additional exposure variable, to test whether associations were independent. All potential confounder variables were then added to the model that included paternal and maternal negative cognitive styles. Maternal and paternal social class and education were highly correlated so we adjusted for the paternal measures only. We tested whether the magnitude of the association between paternal and adolescent cognitive styles differed to maternal cognitive styles using the ‘test’ command in STATA, which performs a Wald test to compare coefficients. To test whether the association differed between male and female offspring, we tested for an interaction between paternal negative cognitive styles and the sex of the child.

#### The role of offspring depressive symptoms

**Figure 1:**
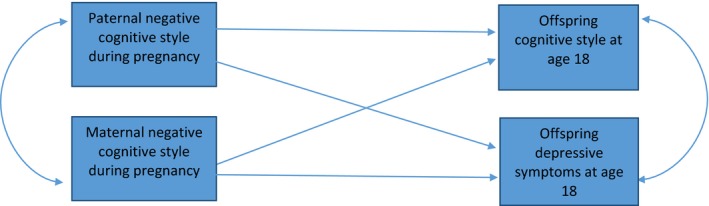
Multivariable structural equation model with offspring cognitive styles and depressive symptoms at age 18 as outcomes, to account for the association between them, and test independent associations with paternal cognitive style. This model was run on multiply imputed data (*N* = 6,123), after adjustment for potential confounders (parent depressive symptoms at 18 weeks gestation, parent social class, parent education, parent age, child gender, whether the parents live together, whether the father is the biological father) [Colour figure can be viewed at http://wileyonlinelibrary.com]

It might be that paternal negative cognitive styles lead to offspring depressive symptoms, and we observe an association with offspring negative cognitive styles because they follow offspring depressive symptoms. To test whether the association between paternal cognitive styles during pregnancy and offspring cognitive styles at age 18 was independent of offspring depressive symptoms at age 18, we used the structural equation modelling (SEM) command in STATA which allows multiple outcomes, Figure 1. This allowed us to control for the association between offspring cognitive styles and offspring depressive symptoms at age 18 (both modelled as continuous outcomes), and test independent associations with paternal cognitive styles. We ran this model on the imputed data using the ‘mi estimate, cmdok: sem’ command, before and after adjustment for confounders (*N* = 6,123). We used the imputed data to increase precision and power, because structural equation models are computationally intensive, especially when multiple pathways are adjusted for multiple confounders and effect sizes are small.

**Figure 2:**
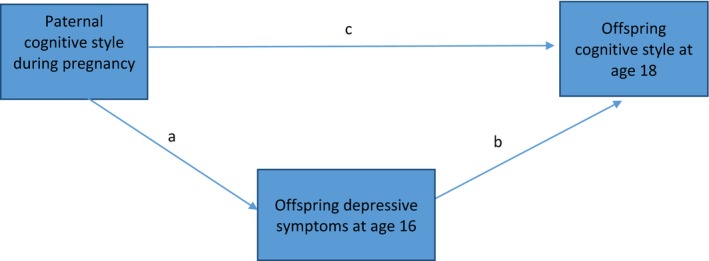
Mediational structural equation model to test the extent to which the association between paternal cognitive styles during pregnancy (exposure) and offspring cognitive styles at age 18 (outcome) is explained (mediated) by offspring depressive symptoms at age 16. This model was run on multiply imputed data (*N* = 6,123), after adjustment for potential confounders (parent depressive symptoms at 18 weeks gestation, parent social class, parent education, parent age, sex of the child, whether the parents live together, whether the father is the biological father) [Colour figure can be viewed at http://wileyonlinelibrary.com]

Any association we observe between paternal and offspring cognitive styles might also be explained by the influence of paternal cognitive styles on earlier offspring depressive symptoms (prior to age 18, when our main outcome measure was assessed). To investigate this, we used the SEM command to test the extent to which offspring depressive symptoms at age 16 mediated the association between paternal cognitive styles during pregnancy and offspring cognitive styles at age 18, Figure 2. We selected age 16 because the prevalence of depression has increased by this age, which also predated our exposure variable (Joinson, Kounali, & Lewis, 2017). We ran this model on the imputed data set using the ‘mi estimate, cmdok: sem’ command. Estimates produced are unstandardized regression coefficients, 95% confidence intervals and *p* values. First, we used the SEM command to estimate the ‘total effect’ (i.e. the association we would observe if there were no mediator) for the association between paternal and offspring cognitive styles. Next, we simultaneously calculated associations between paternal cognitive styles and offspring depressive symptoms at age 16 (path a, Figure 2) and offspring depressive symptoms and cognitive styles (path b, Figure 2). We manually calculated the ‘indirect effect’ (the amount of the total effect that passes through the mediator) by multiplying the coefficients for paths a and b in Figure 2. We then calculated the proportion of the total effect mediated by dividing the indirect effect by the total effect. All pathways in Figure 2 were simultaneously adjusted for potential confounders (see Figure 2).

#### Missing data

Our primary analyses were conducted on an imputed data set (*N* = 6,123) based on those with complete data on the exposure variable, paternal cognitive styles at 18 weeks pregnancy. For comparison, we also present results on the complete case sample (*N* = 1,881). The imputed sample was selected for the primary analyses due to the substantial amount of missing data in ALSPAC from birth to 18 years and because complete case analyses make the unrealistic assumption that data are missing completely at random, that we know is not true in ALSPAC. We also expected a small effect size based on our previous analyses of maternal cognitive styles. In addition to reducing biases due to attrition, the imputed sample would increase statistical power.

We used multiple imputation by chained equations (MICE) to replace missing data. We assumed missingness was dependent on observed data (missing at random) and imputed 50 data sets. To predict missing data, we used all variables selected for the analysis models, all potential confounders (Table 1), previous measures of depressive symptoms collected from age 10 to 18, and a number of auxiliary variables such as parity, and smoking during pregnancy. Analyses were then run across imputed data sets using the ‘mi estimate’ command which fits a model to each of the imputed data sets and pools individual results using Rubin's combination rules (Sterne et al., 2009; White, Royston, & Wood, 2011). We imputed up to the core singleton ALSPAC sample that had complete data on the exposure (paternal cognitive styles), and at least one measure of depressive symptoms collected prior to or at the same time as the outcome (*N* = 6,123).

**Table 1:** Characteristics of the complete case sample, compared to the rest of Avon Longitudinal Study of Parents and Children (ALSPAC)

Characteristic	ALSPAC sample^a^ (*N* = 12,075)	Complete case sample (*N* = 1,881)	*p* value
Female offspring, *N* (%)	5,680 (47.0)	1,064 (56.6)	<.0001
Father not biological father, *N* (%)	54 (0.8)	7 (0.4)	.042
Parents do not live together, *N* (%)	765 (6.9)	41 (2.2)	<.0001
Paternal education O Level or less, *N* (%)	4,406 (55.8)	729 (38.8)	<.0001
Maternal education O Level or less, *N* (%)	7,118 (68.2)	850 (45.2)	<.0001
Lower paternal social class, *N* (%)	4,312 (51.6)	600 (31.9)	<.0001
Lower maternal social class, *N* (%)	1,761 (21.7)	224 (11.0)	<.0001
Paternal smoking during pregnancy, *N* (%)	3,047 (40.2)	462 (25.7)	<.0001
Maternal smoking during pregnancy, *N* (%)	3,006 (26.8)	232 (12.4)	<.0001
Offspring depression diagnoses at age 18, *N* (%)	214 (8.6)	120 (6.9)	.041
Paternal age, Mean (*SD*)	30.1 (5.8)	31.5 (5.4)	<.0001
Maternal age, Mean (*SD*)	27.5 (5.0)	29.3 (4.4)	<.0001
Paternal depression score at baseline, Mean (*SD*)	4.3 (4.0)	3.8 (3.6)	<.0001
Maternal depression score at baseline, Mean (*SD*)	7.2 (4.9)	6.0 (4.4)	<.0001
Paternal cognitive style score at baseline, Mean (*SD*)	4.5 (3.2)	4.3 (3.3)	.0036
Maternal cognitive style score at baseline, Mean (*SD*)	5.0 (3.7)	4.9 (3.5)	.6286
Child cognitive style score at 18, Mean (*SD*)	161.2 (19.7)	162.3 (20.6)	.0928
Child depressive symptoms at 18, Mean (*SD*)	3.3 (4.2)	3.1 (3.7)	.0577

a For core singleton ALSPAC sample not in complete case sample.

## Results

**Figure 3:**
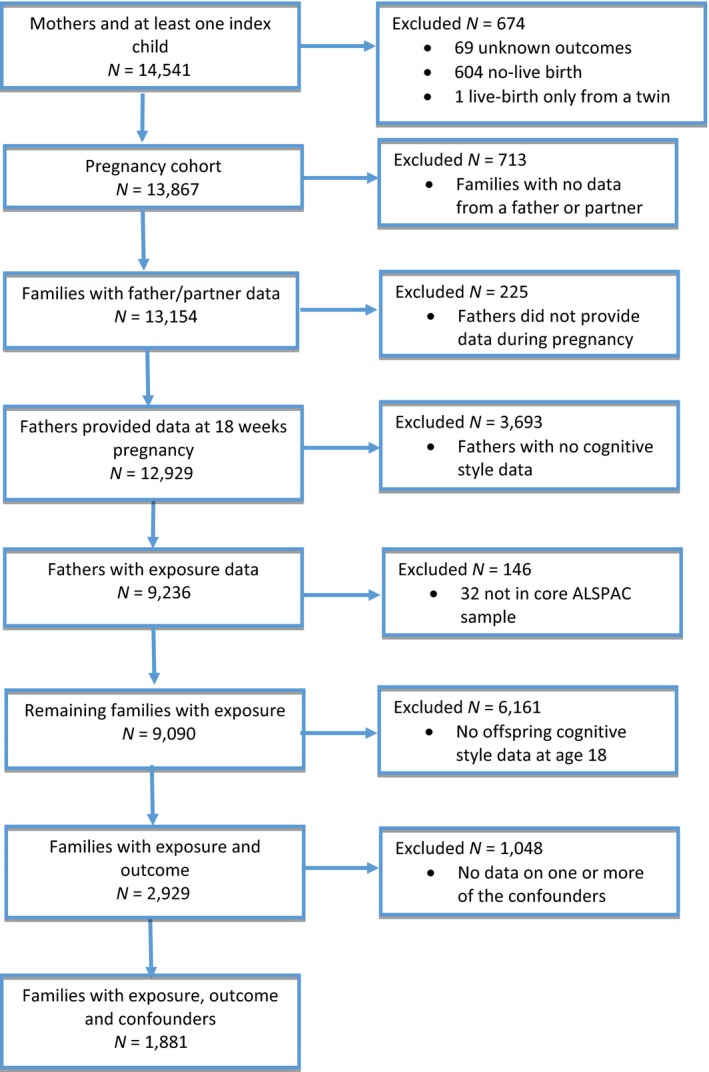
Flow chart of study sample [Colour figure can be viewed at http://wileyonlinelibrary.com]

Figure 3 shows the flow of participants through the sample. Data on paternal negative cognitive styles were available for 9,236 of 12,929 fathers (71%). Data on offspring negative cognitive styles 18 years later were available from 2,929 offspring who also had data on paternal cognitive styles. Once all potential confounders were included, this sample was reduced to 1,881. Demographic characteristics for fathers and offspring with complete data compared to those from the entire ALSPAC cohort are presented in Table 1. Compared to the entire ALSPAC cohort, families with complete data tended to be from higher social classes, were more educated, and had lower parental depressive symptom scores. Characteristics of the sample with complete exposure data (the analytic sample) are presented in Table 2, according to paternal negative cognitive styles (split at the median).

**Table 2:** Characteristics of the sample with complete exposure data, according to paternal negative style (split at the median)

Characteristic	Paternal negative cognitive style		*p* value
	Below median (*N *=* *3,426, 56%)	Above median (*N *=* *2,697, 44%)	
Female offspring, *N* (%)	1,742 (51.2)	1,388 (51.1)	.942
Father not biological father, *N* (%)	11 (0.4)	16 (0.7)	.112
Parents do not live together, *N* (%)	84 (2.5)	98 (3.7)	.010
Paternal education O Level or less, *N* (%)	1,631 (48.2)	1,182 (43.8)	.001
Maternal education O Level or less, *N* (%)	1,916 (57.7)	1,422 (53.5)	.001
Lower paternal social class, *N* (%)	1,231 (41.8)	915 (38.4)	.012
Lower maternal social class, *N* (%)	441 (15.2)	383 (16.3)	.238
Paternal smoking during pregnancy, *N* (%)	1,053 (32.2)	835 (32.5)	.812
Maternal smoking during pregnancy, *N* (%)	587 (17.3)	457 (16.9)	.661
Offspring depression diagnoses at age 18, *N* (%)	128 (7.4)	112 (7.7)	.727
Paternal age, Mean (*SD*)	30.8 (5.4)	31.3 (5.6)	.0003
Maternal age, Mean (*SD*)	28.5 (4.4)	28.8 (4.6)	.0106
Paternal depression score at baseline, Mean (*SD*)	2.9 (3.1)	5.5 (4.1)	<.0001
Maternal depression score at baseline, Mean (*SD*)	6.0 (4.5)	6.9 (4.6)	<.0001
Maternal cognitive style score at baseline, Mean (*SD*)	4.5 (3.4)	5.5 (3.5)	<.0001
Child depressive symptoms at 18, Mean (*SD*)	3.1 (3.8)	3.4 (3.9)	.0124

### The association between paternal and offspring negative cognitive styles, and the role of maternal negative cognitive styles

Unadjusted and adjusted associations between paternal and offspring cognitive styles are presented in Table 2, for the imputed sample (*N* = 6,123). There was evidence of a positive association between paternal and offspring cognitive styles; a 6‐point (two standard deviations) increase in paternal cognitive style scores during pregnancy was associated with a 2.86‐point (0.1 of a standard deviation) increase in offspring cognitive style scores at 18 years of age (95% CI 1.36–4.37). This association remained after maternal cognitive styles, which were weakly associated with paternal cognitive styles (*r* = .14, *p* < .001), were added to the model. The association also remained after adjustment for paternal depressive symptoms and other potential confounders (adjusted change in offspring cognitive style scores: 1.92, 95% CI 0.20–3.64). Results from the complete case sample were very similar, Table 3. We found no evidence for an interaction between paternal negative cognitive style and the sex of the child (*p* = .899), suggesting that the association was similar for females and males. We found no evidence that the magnitude of the association between paternal and offspring cognitive styles differed to the association with maternal cognitive styles *F*(1, 1867) = 0.10, *p* = .7511.

**Table 3:** Change in offspring negative cognitive style scores at age 18 for each 6‐point (approximately two standard deviations) increase in paternal negative cognitive style scores during pregnancy, imputed sample (*N *=* *6,123)

Model	Change in offspring negative cognitive styles (95% CI) *p* value
Model 1: Univariable association	2.86 (1.36–4.37) <.0001
Model 2: Model 1 adjusted for maternal cognitive style	2.40 (0.87–3.92) .002
Model 3: Model 2 adjusted for paternal depressive symptoms	2.22 (0.50–3.94) .012
Model 4: Model 3 adjusted for other confounders^a^	1.92 (0.20–3.64) .029

a Confounders were: maternal depressive symptoms at 18 weeks gestation, parental social class, parental education, parental age, child gender, whether the parents live together, whether the father is the biological father.

### The role of offspring depressive symptoms

Controlling for the association between offspring cognitive styles and offspring depressive symptoms at age 18 resulted in a very similar pattern of associations between paternal and offspring cognitive styles, Table 4. The fully adjusted change in offspring cognitive style scores for a 6‐point increase in paternal cognitive style scores was 1.92 (95% CI 0.20–3.63). In the unadjusted multivariable model, we also found evidence for a positive association between paternal negative cognitive style and offspring depressive symptoms (.29, 95% CI 0.04 to 0.54). However, this attenuated after adjustment for confounders (.17, 95% CI −0.12 to 0.45), Table 5.

**Table 4:** Change in offspring negative cognitive style scores at age 18 for each 6‐point (approximately two standard deviations) increase in paternal negative cognitive style scores during pregnancy, complete case sample (*N* = 1,881)

Model	Change in offspring negative cognitive styles (95% CI) *p* value
Model 1: Univariable association	3.33 (1.56–5.11) <.0001
Model 2: Model 1 adjusted for maternal cognitive style	2.93 (1.14–4.72) .001
Model 3: Model 2 adjusted for paternal depressive symptoms	2.84 (0.86–4.82) .005
Model 4: Model 3 adjusted for other confounders^a^	2.55 (0.56–4.54) .012

a Confounders were: maternal depressive symptoms at 18 weeks gestation, parental social class, parental education, parental age, child gender, whether the parents live together, whether the father is the biological father.

**Table 5:** Multivariable model (two outcomes): change in offspring negative cognitive styles (outcome 1) and offspring depressive symptoms (outcome 2) at age 18 for each 6‐point (approximately two standard deviations) increase in paternal negative cognitive style scores during pregnancy, imputed sample (*N* = 6,123)

Model	Change in offspring cognitive styles (95% CI) *p* value	Change in offspring depressive symptoms (95% CI) *p* value
Model 1: Univariable association	2.86 (1.36 to 4.37) <.0001	0.29 (0.04 to 0.54) .025
Model 2: Model 1 adjusted for maternal cognitive style	2.40 (0.87 to 3.92) .002	0.21 (−0.05 to 0.46) .115
Model 3: Model 2 adjusted for paternal depressive symptoms	2.22 (0.50 to 3.94) .012	0.14 (−0.15 to 0.43) .333
Model 4: Model 3 adjusted for other confounders^a^	1.92 (0.20 to 3.63) .029	0.17 (−0.12 to 0.45) .251

a Confounders were: maternal depressive symptoms at 18 weeks gestation, parental social class, parental education, parental age, child gender, whether the parents live together, whether the father is the biological father.

Using the SEM command, the estimated ‘total effect’ for the association between paternal and offspring cognitive styles was 1.92 (95% CI 0.20–3.64) (after adjustment for confounders). After adjustments for confounders, we found strong evidence that paternal negative cognitive styles during pregnancy were associated with offspring depressive symptoms at age 16; a 6‐point increase in paternal negative cognitive style scores was associated with an increase in .59 of a MFQ point (95% CI 0.24–0.93). In turn, there was strong evidence that offspring depressive symptoms at age 16 were associated with their cognitive styles at age 18 (.99, 95% CI 0.84–1.14). We found evidence that 20% of the association between paternal cognitive styles during pregnancy and offspring cognitive styles at age 18 was mediated through offspring depressive symptoms at age 16. Evidence remained for a direct effect of paternal cognitive styles on offspring cognitive styles (1.52, 95% CI 0.02–3.02, *p* value .047).

## Discussion

We found evidence for an association between paternal negative cognitive styles, assessed when mothers were 18 weeks pregnant, and offspring negative cognitive styles 18 years later. A 6‐point increase in paternal negative cognitive style was associated with around a 2‐point increase in offspring negative cognitive style. Although small, this effect size did not differ to that reported for maternal and offspring negative cognitive styles in our previous study of this sample (Pearson et al., 2013). It is notable that we observed an association between paternal and offspring cognitive styles 18 years later, given the number of different influences on children's development during this period, and the possible reduction in parental influences as children mature. Consistent with our previous study, the association between paternal and offspring negative cognitive styles was independent of depression in both the parents and the offspring. It was also independent of maternal negative cognitive style, suggesting that mothers and fathers both independently contribute to the development of negative cognitive styles in their offspring.

### Strengths and limitations

To our knowledge this is the largest study to examine the association between paternal and offspring negative cognitive styles, and the first to do so in a longitudinal sample recruited from the general population. Our sample was a population‐based birth cohort followed from pregnancy to early adulthood, which allowed us to examine long‐term associations between paternal and offspring cognitive styles. Because data on paternal cognitive styles were collected before the child was born, this also eliminates the possibility of reverse causation.

A limitation of our study was loss to follow‐up over the 18‐year period. Our complete case sample differed to the overall ALSPAC sample; they tended to come from more educated families, had fewer parental depressive symptoms and fewer offspring depression diagnoses. Paternal cognitive style scores were also slightly higher in the complete case sample. Data were therefore not ‘missing completely at random’. When data are not ‘missing completely at random’, complete case analyses can introduce bias (Sterne et al., 2009). Also, due to the cumulative effect of missing data in several variables, the complete case sample size was reduced enormously, leading to a substantial loss of power and precision (Sterne et al., 2009). We used multiple imputation to replace missing data and the wealth of data available in ALSPAC allowed us to identify a number of variables that were associated with missing data, supporting the plausibility of the missing at random assumption. Results from our imputed sample were consistent with those from our complete cases analysis, suggesting that missing data did not substantially bias our results.

Another limitation was that we used different measures to assess negative cognitive styles in fathers and offspring. The paternal measure was based on the Dysfunctional Attitudes Scale (DAS) whilst for offspring we used the CSQ. However, as stated in the introduction, the DAS and CSQ are correlated with each other and also have a conceptual overlap (Alloy et al., 2001; Pearson et al., 2014). Furthermore, as paternal cognitive styles were measured before the child was born, any measurement error in the exposure is likely to be unrelated to measurement error in the outcome and could not be biased by the outcome.

**Figure 4:**
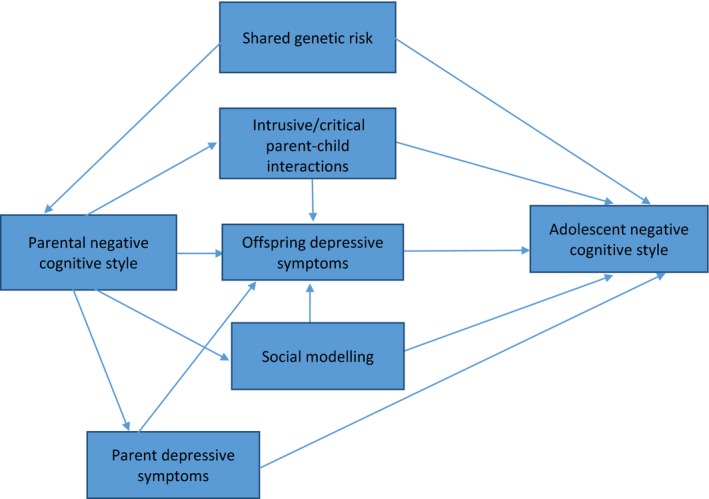
Directed acyclic graph (DAG) displaying hypothesized mechanisms that might underlie the association between parental negative cognitive styles in childhood and offspring negative cognitive styles in adolescence [Colour figure can be viewed at http://wileyonlinelibrary.com]

Although we adjusted for several potential confounders, as with all observational studies, residual confounding is still a possibility. The wealth of detailed data in the ALSPAC cohort, however, meant that a range of potential confounders were available, and none of these appeared to account for our association. We also controlled for depressive symptoms at age 18 in the offspring which led to an attenuation of the strength of our association. However, it is important to note that this attenuation might have occurred because offspring depressive symptoms are part of the causal pathway between paternal and offspring negative cognitive styles. Finally, the data available in ALSPAC did not allow us to test more detailed mechanisms that might account for the association between paternal and offspring negative cognitive styles (Figure 4). However, while understanding mechanisms is important, it is less of a pressing concern when the exposure variable itself is modifiable. There is very good evidence that negative cognitive styles can be targeted and modified, and our results suggest that this could reduce the prevalence of negative cognitive styles in offspring. We therefore think that, at this early stage of research into the development of negative cognitive styles, detailed investigations of exposure and outcome were required before mechanistic ones, and that targeting the exposure itself would be effective.

### Possible mechanisms underlying our association

Negative cognitive styles are stable and enduring beliefs that persist over time and underlie chronic and recurrent illnesses like depression, unless they are modified (Beck, 1967; Renner, Lobbestael, Peeters, Arntz, & Huibers, 2012). In our sample, the parental negative cognitive styles assessed during pregnancy are assumed to remain relatively stable throughout the child's development. We cannot tell from our study when negative cognitive styles in fathers influenced the development of negative cognitive styles in offspring, as we do not have earlier measures of offspring cognitive styles. However, our results support the hypothesis that this influence begins in early life and persists to adulthood. Future studies with earlier measures of offspring cognitive styles would be of use, to identify possible timing effects that could refine the development of preventive interventions.

There are several specific mechanisms that could account for our association. First, it is possible that negative cognitive styles are genetically transmitted, since the vast majority of fathers in ALSPAC (99%) are biological fathers. However, twin studies report that, although genetic influences might contribute to associations between paternal and offspring cognitive styles, environmental processes are more important (Lau & Eley, 2008; Lau, Rijsdijk, & Eley, 2006). For example, one study of an adolescent sample found that 35% of the variance in cognitive style scores could be attributed to genetic influences, the rest to the environment (Lau et al., 2006). There is also evidence that, environmental as well as genetic processes are important in the intergenerational transmission of depression (Lewis, Rice, Harold, Collishaw, & Thapar, 2011).

A second possible mechanism is social learning or modelling. Offspring might develop their own negative cognitive style by modelling it from their parents. Studies of people with depression suggest that negative cognitive styles are expressed, for example, in conversation (Goodman & Gotlib, 1999). This supports the hypothesis that the cognitive styles of fathers would be sufficiently expressed and observed by children, for them to acquire through social modelling. The equivalence of the maternal and paternal associations in our sample implies that, if social modelling is the mechanism, the influence of the mother and father as ‘models’ is largely equivalent.

Parents who think more negatively about themselves might also interpret their child's experiences more negatively. For example, if a child fails an exam, a parent with a more negative cognitive style might attribute this failure to their own failures as a parent, or to the child. Parents who think more negatively about themselves and their circumstances might also be more critical or intrusive in how they interact with their children, which can lead to higher levels of offspring self‐criticalness (Hong et al., 2017). An absence of parental modelling of positive ways of coping with stressors might also lead to the development of negative cognitive styles in offspring.

Another possible mechanism is parenting style. For example, fathers with depression experience more conflict with their offspring (Nath, Russell, Ford, Kuyken, & Psychogiou, 2015), and have been found to engage in less positive parenting behaviours (Wilson & Durbin, 2010). Although we adjusted for paternal depression in our analyses, similar mechanisms might occur in fathers who have more negative cognitive styles. Further research on these mechanisms, how they operate and how they might be prevented is required.

Reliable measures of social modelling and critical or conflictual parent–child interactions are unavailable in the ALSPAC data set, so we were unable to explore these mechanisms. If mechanisms of the family environment are important in the intergenerational transmission of paternal negative cognitive styles, one hypothesis is that the association would be stronger in families where the father and child live together. We were also unable to explore this possibility, due to the small number of fathers who provided data on their cognitive styles but did not live with the mother during her pregnancy. However, this is an important direction for future research. This might also cast light on the relative importance of genetic influences. If associations do not differ in magnitude between fathers who do and do not live at home, this could suggest a role for genetic influences.

### Implications

Our findings are important because adolescents with more negative cognitive styles are at increased risk of future depression, and negative cognitive styles are modifiable (Evans et al., 2005; Haeffel et al., 2003; Hankin, 2008; Pearson et al., 2014). It is therefore possible that preventing the development of negative cognitive styles could also prevent depression. Second, our findings demonstrate that fathers’ thoughts and beliefs about themselves and their circumstances are as important as mothers’, to the development of negative thinking styles among offspring. This is important to acknowledge given that fathers are increasingly involved in the care of their children in many countries (Haas & Hwang, 2013). It is therefore important that interventions acknowledge the role of paternal influences.

Our findings raise the possibility that modifying negative cognitive styles might prevent depression, although further research in this area would be required to test this. In principle, parents could directly modify their negative cognitive styles using CBT. CBT targets negative thoughts and beliefs, so theoretically it should address negative cognitive styles even when people are not depressed, although we are not aware of any evidence that this is effective. CBT can also be delivered in different modalities, including online. Online delivery has been found to be effective irrespective of the severity of depressive symptoms (Hallgren et al., 2015; Jarrett, Vittengl, Doyle, & Clark, 2007).

Instead of modifying cognitive styles directly, parents could also reduce negative expressions in the presence of their children. For example, parents could modify the negative ways that they might speak about themselves or their children, in front of their children. This would require some knowledge of what a negative expression/cognitive style is. One of the ways that this could be achieved is via the use of parenting interventions. There are many family‐based interventions that target parent–child interactions, but few that also consider the expression of negative thinking styles by both parents. Our study highlights, for the first time, the potential importance of targeting negative thinking styles in mothers and fathers. Our findings support the inclusion of fathers in parenting interventions designed to improve parent–child relationships and/or prevent offspring depression. Parenting interventions, including those for mothers and fathers with depression, could therefore put more emphasis on encouraging fathers to reduce their negative self‐expressions. A key contribution of our study is that including fathers in preventative family‐based interventions could be as important as including mothers.

**Key points**

- *What is known:* Preventing the development of negative thinking styles could prevent depression. Maternal negative cognitive styles during pregnancy increase the risk of offspring negative cognitive styles in adolescence. However, evidence on paternal cognitive styles is inconclusive and there have only been a few small studies.
- *What is new:* We conducted the first large longitudinal study of paternal and adolescent negative cognitive styles. We found strong evidence for an association between paternal negative cognitive styles, measured during the mother's pregnancy, and negative cognitive styles in offspring at 18 years of age. The association was independent of depression in both parents and offspring, and the effect size was equivalent to that reported for maternal depressogenic cognitive styles in our previous study of this sample.
- *What is clinically relevant:* Fathers should be included in preventative family based interventions to prevent the development of adolescent negative thinking styles, and possibly depression.

## Supporting information


**Table S1.** Change in offspring negative cognitive style scores at age 18 for one standard deviation increase in paternal negative cognitive style scores during pregnancy, imputed sample (*N* = 6,123), standardized regression coefficients.

## References

[jcpp12847-bib-0001] Abramson, L.Y. , Seligman, M.E. , & Teasdale, J.D. (1978). Learned helplessness in humans: Critique and reformulation. Journal of Abnormal Psychology, 87, 49–74. 649856

[jcpp12847-bib-0002] Alloy, L.B. , Abramson, L.Y. , Metalsky, G.I. , & Hartlage, S. (1988). The hopelessness theory of depression: Attributional aspects. The British Journal of Clinical Psychology/The British Psychological Society, 27(Pt 1), 5–21. 10.1111/j.2044-8260.1988.tb00749.x 3281732

[jcpp12847-bib-0003] Alloy, L.B. , Abramson, L.Y. , Tashman, N.A. , Berrebbi, D.S. , Hogan, M.E. , Whitehouse, W.G. , … & Morocco, A. (2001). Developmental origins of cognitive vulnerability to depression: Parenting, cognitive, and inferential feedback styles of the parents of individuals at high and low cognitive risk for depression. Cognitive Therapy and Research, 25, 397–423.

[jcpp12847-bib-0004] Alloy, L.B. , Abramson, L.Y. , Whitehouse, W.G. , Hogan, M.E. , Panzarella, C. , & Rose, D.T. (2006). Prospective incidence of first onsets and recurrences of depression in individuals at high and low cognitive risk for depression. Journal of Abnormal Psychology, 115, 145–156. 16492105 10.1037/0021-843X.115.1.145

[jcpp12847-bib-0005] Angold, A. , Costello, E. , Messer, S. , Pickles, A. , Winder, F. , & Silver, D. (1995). The development of a short questionnaire for use in epidemiological studies of depression in children and adolescents. International Journal of Methods in Psychiatric Research, 5, 237–249.

[jcpp12847-bib-0006] Beck, A. (1967). Depression: Clinical, experimental, and theoretical aspects. New York: Harper & Row.

[jcpp12847-bib-0007] Beck, A. (2008). The evolution of the cognitive model of depression and its neurobiological correlates. American Journal of Psychiatry, 165, 969–977. 18628348 10.1176/appi.ajp.2008.08050721

[jcpp12847-bib-0008] Boyd, A. , Golding, J. , Macleod, J. , Lawlor, D.A. , Fraser, A. , Henderson, J. , … & Davey Smith, G. (2013). Cohort Profile: The “children of the 90s”–the index offspring of the Avon Longitudinal Study of Parents and Children. International Journal of Epidemiology, 42, 111–127. 22507743 10.1093/ije/dys064 PMC3600618

[jcpp12847-bib-0009] Carothers, A. , & Murray, L. (1990). The Validation of the Edinburgh Post‐natal Depression Scale on a community sample. British Journal of Psychiatry, 157, 288–290. 2224383 10.1192/bjp.157.2.288

[jcpp12847-bib-0010] Cuijpers, P. , Cristea, I.A. , Karyotaki, E. , Reijnders, M. , & Huibers, M.J.H. (2016). How effective are cognitive behavior therapies for major depression and anxiety disorders? A meta‐analytic update of the evidence. World Psychiatry, 15, 245–258. 27717254 10.1002/wps.20346 PMC5032489

[jcpp12847-bib-0011] Edmondson, O.J.H. , Psychogiou, L. , Vlachos, H. , Netsi, E. , & Ramchandani, P.G. (2010). Depression in fathers in the postnatal period: Assessment of the Edinburgh Postnatal Depression Scale as a screening measure. Journal of Affective Disorders, 125, 365–368. 20163873 10.1016/j.jad.2010.01.069 PMC2927780

[jcpp12847-bib-0012] Evans, J. , Heron, J. , Lewis, G. , Araya, R. , Wolke, D. , & ALSPAC Study Team . (2005). Negative self‐schemas and the onset of depression in women: Longitudinal study. The British Journal of Psychiatry: The Journal of Mental Science, 186, 302–307. 15802686 10.1192/bjp.186.4.302

[jcpp12847-bib-0013] Ferrari, A.J. , Somerville, A.J. , Baxter, A.J. , Norman, R. , Patten, S.B. , Vos, T. , & Whiteford, H.A. (2013). Global variation in the prevalence and incidence of major depressive disorder: A systematic review of the epidemiological literature. Psychological Medicine, 43, 471–481. 22831756 10.1017/S0033291712001511

[jcpp12847-bib-0014] Goodman, S.H. , & Gotlib, I.H. (1999). Risk for psychopathology in the children of depressed mothers: A developmental model for understanding mechanisms of transmission. Psychological Review, 106, 458–490. 10467895 10.1037/0033-295x.106.3.458

[jcpp12847-bib-0015] Haas, L. , & Hwang, P. (2013). Fatherhood and social policy in Scandinavia. New York: Routledge/Taylor & Francis Group.

[jcpp12847-bib-0016] Haeffel, G.J. , Abramson, L.Y. , Voelz, Z.R. , Metalsky, G.I. , Halberstadt, L. , Dykman, B.M. , … & Alloy, L.B. (2003). Cognitive vulnerability to depression and lifetime history of Axis I psychopathology: A comparison of negative cognitive styles (CSQ) and dysfunctional attitudes (DAS). Journal of Cognitive Psychotherapy, 17, 3–22.

[jcpp12847-bib-0017] Haeffel, G.J. , Gibb, B.E. , Metalsky, G.I. , Alloy, L.B. , Abramson, L.Y. , Hankin, B.L. , … & Swendsen, J.D. (2008). Measuring cognitive vulnerability to depression: Development and validation of the cognitive style questionnaire. Clinical Psychology Review, 28, 824–836. 18234405 10.1016/j.cpr.2007.12.001 PMC4090011

[jcpp12847-bib-0018] Hallgren, M. , Kraepelien, M. , Öjehagen, A. , Lindefors, N. , Zeebari, Z. , Kaldo, V. , & Forsell, Y. (2015). Physical exercise and internet‐based cognitive–behavioural therapy in the treatment of depression: Randomised controlled trial. The British Journal of Psychiatry, 207, 227–234. 26089305 10.1192/bjp.bp.114.160101

[jcpp12847-bib-0019] Hankin, B.L. (2008). Cognitive vulnerability‐stress model of depression during adolescence: Investigating depressive symptom specificity in a multi‐wave prospective study. Journal of Abnormal Child Psychology, 36, 999–1014. 18437551 10.1007/s10802-008-9228-6 PMC2763423

[jcpp12847-bib-0020] Hankin, B.L. , & Abramson, L.Y. (2002). Measuring cognitive vulnerability to depression in adolescence: Reliability, validity, and gender differences. Journal of Clinical Child & Adolescent Psychology, 31, 491–504. 12402568 10.1207/S15374424JCCP3104_8

[jcpp12847-bib-0021] Hong, R.Y. , Lee, S.S.M. , Chng, R.Y. , Zhou, Y. , Tsai, F.‐F. , & Tan, S.H. (2017). Developmental trajectories of maladaptive perfectionism in middle childhood. Journal of Personality, 85, 409–422. 26919690 10.1111/jopy.12249

[jcpp12847-bib-0022] Jarrett, R.B. , Vittengl, J.R. , Doyle, K. , & Clark, L.A. (2007). Changes in cognitive content during and following cognitive therapy for recurrent depression: Substantial and enduring, but not predictive of change in depressive symptoms. Journal of Consulting and Clinical Psychology, 75, 432–446. 17563160 10.1037/0022-006X.75.3.432 PMC2605093

[jcpp12847-bib-0023] Joinson, C. , Kounali, D. , & Lewis, G. (2017). Family socioeconomic position in early life and onset of depressive symptoms and depression: A prospective cohort study. Social Psychiatry and Psychiatric Epidemiology, 52, 95–103. 27837235 10.1007/s00127-016-1308-2 PMC5226994

[jcpp12847-bib-0024] Kaslow, N.J. , Rehm, L.P. , Pollack, S.L. , & Siegel, A.W. (1988). Attributional style and self‐control behavior in depressed and nondepressed children and their parents. Journal of Abnormal Child Psychology, 16, 163–175. 3385081 10.1007/BF00913592

[jcpp12847-bib-0025] Kroll, M.E. , Carson, C. , Redshaw, M. , & Quigley, M.A. (2016). Early father involvement and subsequent child behaviour at ages 3, 5 and 7 years: Prospective analysis of the UK millennium cohort study. PLoS ONE, 11, e0162339. 27654635 10.1371/journal.pone.0162339 PMC5031314

[jcpp12847-bib-0026] Lakdawalla, Z. , Hankin, B.L. , & Mermelstein, R. (2007). Cognitive theories of depression in children and adolescents: A conceptual and quantitative review. Clinical Child and Family Psychology Review, 10, 1–24. 17318382 10.1007/s10567-006-0013-1

[jcpp12847-bib-0027] Lau, J.Y.F. , & Eley, T.C. (2008). Attributional style as a risk marker of genetic effects for adolescent depressive symptoms. Journal of Abnormal Psychology, 117, 849–859. 19025231 10.1037/a0013943

[jcpp12847-bib-0028] Lau, J.Y.F. , Rijsdijk, F. , & Eley, T.C. (2006). I think, therefore I am: A twin study of attributional style in adolescents. Journal of Child Psychology and Psychiatry, 47, 696–703. 16790004 10.1111/j.1469-7610.2005.01532.x

[jcpp12847-bib-0029] Lewis, G. , Pelosi, A.J. , Araya, R. , & Dunn, G. (2009). Measuring psychiatric disorder in the community: A standardized assessment for use by lay interviewers. Psychological Medicine, 22, 465–486. 10.1017/s0033291700030415 1615114

[jcpp12847-bib-0030] Lewis, G. , Rice, F. , Harold, G.T. , Collishaw, S. , & Thapar, A. (2011). Investigating environmental links between parent depression and child depressive/anxiety symptoms using an assisted conception design. Journal of the American Academy of Child and Adolescent Psychiatry, 50, 451–459. 21515194 10.1016/j.jaac.2011.01.015 PMC3136241

[jcpp12847-bib-0031] McManus, S. , Bebbington, P. , Jenkins, R. , & Brugha, T.S. (2016). Mental health and well‐being in England: Adult Psychiatric Morbidity Survey 2014. Leeds: NHS Digital.

[jcpp12847-bib-0032] Meins, E. , McCarthy‐Jones, S. , Fernyhough, C. , Lewis, G. , Bentall, R.P. , & Alloy, L.B. (2012). Assessing negative cognitive style: Development and validation of a Short‐Form version of the Cognitive Style Questionnaire. Personality and Individual Differences, 52, 581–585. 22389545 10.1016/j.paid.2011.11.026 PMC3289144

[jcpp12847-bib-0033] Nath, S. , Russell, G. , Ford, T. , Kuyken, W. , & Psychogiou, L. (2015). Postnatal paternal depressive symptoms associated with fathers’ subsequent parenting: Findings from the Millennium Cohort Study. British Journal of Psychiatry, 207, 558–559. 26494871 10.1192/bjp.bp.114.148379

[jcpp12847-bib-0034] Oliver, J. , & Berger, L. (1992). Depression, parent‐offspring relationships, and cognitive vulnerability. Journal of Social Behavior and Personality, 7, 415–429.

[jcpp12847-bib-0035] Pearson, R.M. , Fernyhough, C. , Bentall, R. , Evans, J. , Heron, J. , Joinson, C. , … & Lewis, G. (2013). Association between maternal depressogenic cognitive style during pregnancy and offspring cognitive style 18 years later. American Journal of Psychiatry, 170, 434–441. 23318526 10.1176/appi.ajp.2012.12050673 PMC3640292

[jcpp12847-bib-0036] Pearson, R.M. , Heron, J. , Button, K. , Bentall, R.P. , Fernyhough, C. , Mahedy, L. , … & Lewis, G. (2014). Cognitive styles and future depressed mood in early adulthood: The importance of global attributions. Journal of Affective Disorders, 171C, 60–67. 10.1016/j.jad.2014.08.057 PMC4222738 25285900

[jcpp12847-bib-0037] Rawal, A. , Collishaw, S. , Thapar, A. , & Rice, F. (2013). A direct method of assessing underlying cognitive risk for adolescent depression. Journal of Abnormal Child Psychology, 41, 1279–1288. 23702578 10.1007/s10802-013-9760-x

[jcpp12847-bib-0038] Renner, F. , Lobbestael, J. , Peeters, F. , Arntz, A. , & Huibers, M. (2012). Early maladaptive schemas in depressed patients: Stability and relation with depressive symptoms over the course of treatment. Journal of Affective Disorders, 136, 581–590. 22119093 10.1016/j.jad.2011.10.027

[jcpp12847-bib-0039] Seligman, M.E. , Peterson, C. , Kaslow, N.J. , Tanenbaum, R.L. , Alloy, L.B. , & Abramson, L.Y. (1984). Attributional style and depressive symptoms among children. Journal of Abnormal Psychology, 93, 235–238. 6725758 10.1037//0021-843x.93.2.235

[jcpp12847-bib-0040] Stark, K.D. , Schmidt, K.L. , & Joiner, T.E. (1996). Cognitive triad: Relationship to depressive symptoms, parents’ cognitive triad, and perceived parental messages. Journal of Abnormal Child Psychology, 24, 615–631. 8956087 10.1007/BF01670103

[jcpp12847-bib-0041] Sterne, J.A.C. , White, I.R. , Carlin, J.B. , Spratt, M. , Royston, P. , Kenward, M.G. , … & Carpenter, J.R. (2009). Multiple imputation for missing data in epidemiological and clinical research: Potential and pitfalls. BMJ (Clinical Research ed.), 338, b2393. 10.1136/bmj.b2393 PMC2714692 19564179

[jcpp12847-bib-0042] Tilling, K. , Macdonald‐Wallis, C. , Lawlor, D.A. , Hughes, R.A. , & Howe, L.D. (2014). Modelling childhood growth using fractional polynomials and linear splines. Annals of Nutrition & Metabolism, 65, 129–138. 25413651 10.1159/000362695 PMC4264511

[jcpp12847-bib-0043] Turk, E. , & Bry, B.H. (1992). Adolescents’ and parents’ explanatory styles and parents’ causal explanations about their adolescents. Cognitive Therapy and Research, 16, 349–357.

[jcpp12847-bib-0044] White, I.R. , Royston, P. , & Wood, A.M. (2011). Multiple imputation using chained equations: Issues and guidance for practice. Statistics in Medicine, 30, 377–399. 21225900 10.1002/sim.4067

[jcpp12847-bib-0045] Wilson, S. , & Durbin, C.E. (2010). Effects of paternal depression on fathers’ parenting behaviors: A meta‐analytic review. Clinical Psychology Review, 30, 167–180. 19926376 10.1016/j.cpr.2009.10.007

