# Supplementary material for: The association between paternal depressogenic cognitive styles during pregnancy and offspring depressogenic cognitive styles: an 18‐year prospective cohort study
Source: J Child Psychol Psychiatry. 2017 Nov 24;59(5):604–14. doi: 10.1111/jcpp.12847 (PMC5947551; doi:10.1111/jcpp.12847)
Supplement: Supplementary file 1 — Table S1. Change in offspring negative cognitive style scores at age 18 for one standard deviation increase in paternal negative cognitive style scores during pregnancy, imputed sample (N = 6,123), standardized regression coefficients. [file JCPP-59-604-s001.docx]

Additional supporting information for: The association between paternal depressogenic cognitive styles during mothers’ pregnancy and offspring depressogenic cognitive styles: an 18-year prospective cohort study – by Gemma Lewis et al.

**Supplementary** **Table S1.** Change in offspring negative cognitive style scores at age 18 for one standard deviation increase in paternal negative cognitive style scores during pregnancy, imputed sample (N=6123), standardized regression coefficients.

| Model | Beta value (95% CI) p value |
| --- | --- |
| Model 1: Univariable association | .07 (.04 to .11) <.0001 |
| Model 2: Model 1 adjusted for maternal cognitive style | .06 (.02 to .10) .002 |
| Model 3: Model 2 adjusted for paternal depressive symptoms | .06 (.01 to .10) .012 |
| Model 4: Model 3 adjusted for other confounders^a^ | .05 (.01 to .09) .029 |

^a^ Confounders were: maternal depressive symptoms at 18 weeks gestation, parental social class, parental education, parental age, child gender, whether the parents live together, whether the father is the biological father.
